# Oligosaccharide Lactate Nanoparticles Enhance Tissue Targeting: A Case Study of the Controlled Delivery of Bedaquiline to Cardiac Tissue in TB Pericarditis

**DOI:** 10.3390/molecules30132845

**Published:** 2025-07-03

**Authors:** Simisola Ayodele, Pradeep Kumar, Armorel van Eyk, Pieter van der Bijl, Yahya E. Choonara

**Affiliations:** 1 Wits Advanced Drug Delivery Platform Research Unit, Department of Pharmacy and Pharmacology, School of Therapeutic Sciences, Faculty of Health Sciences, University of the Witwatersrand, Johannesburg 2193, South Africa; 1715904@students.wits.ac.za (S.A.); pradeep.kumar@wits.ac.za (P.K.); 2 Department of Pharmacy and Pharmacology, School of Therapeutic Sciences, Faculty of Health Sciences, University of the Witwatersrand, Johannesburg 2193, South Africa; armorel.vaneyk@wits.ac.za; 3 Netcare Kuilsriver Hospital, Kuilsriver, 33 Van Riebeeck Road, Kuils River, Cape Town 7580, South Africa; pieter.vanderbijl@gmail.com; 4 Wits Infectious Diseases and Oncology Research Institute, Faculty of Health Sciences, University of the Witwatersrand, Johannesburg 2193, South Africa

**Keywords:** bedaquiline, permeation studies, tuberculosis, polymeric nanosystems, pericardium

## Abstract

Bedaquiline is known to shorten the duration of therapy of tuberculosis but has limitations, e.g., poor solubility and adverse effects such as prolongation of the QT interval. In this study, bedaquiline was incorporated into an inherently targeted nanosystem for improved permeation of the drug, with ex vivo diffusion studies performed to investigate its penetration. The bedaquiline-loaded mannan–chitosan oligosaccharide lactate nanoparticles were prepared by a one-step ionic gelation probe sonication method. A PermeGear 7-in-line flow-through diffusion system was used for the ex vivo diffusion studies across porcine and human pericardia. Bedaquiline-loaded nanoparticles with a particle size and potential of 192.4 nm and 40.5 mV, respectively, were obtained. The drug-loaded mannan–chitosan nanoparticles had an encapsulation efficacy of 98.7% and drug loading of 0.6%. Diffusion data indicated a steady-state flux of 2.889 and 2.346 µg.cm^−2^.min^−1^ for porcine and human pericardia, respectively. The apparent permeability coefficients were calculated to be 2.66 × 10^−4^ cm.min^−1^ and 2.16 × 10^−4^ cm.min^−1^ for porcine and human pericardia, respectively. The lag phases were 52.72 min and 0 min for porcine and human pericardia, respectively. The drug permeation indicated a consistent and linear diffusion pattern across both porcine and human pericardia, additionally approving the porcine pericardium as a great comparable tissue to human tissue for pericardial studies. This study is the first to demonstrate ex vivo diffusion of bedaquiline-loaded, macrophage-targeted chitosan–mannan nanoparticles across both human and porcine pericardia, representing a novel platform for disease-targeted, localized treatment of TB pericarditis.

## 1. Introduction

Tuberculosis is primarily understood as a lung disease, but since the emergence of antimicrobial resistance and long duration of treatment, the *Mycobacterium tuberculosis* (*M.tb*) bacilli has demonstrated pernicious effects via hematological and lymphatic spread outside of the lungs, resulting in a variety of forms of extrapulmonary tuberculosis [1]. Extrapulmonary tuberculosis (EPTB) accounts for 15 to 20% of all TB patients, with a mortality rate as high as 2.16 people per 100 days of hospital stay in a clinical study conducted in Mozambique [2].

Even though extrapulmonary tuberculosis presents such virulence due to drug resistance, only 17 new systemic antibiotics have been approved by the FDA since 2010, amongst which only 2 were registered for drug-resistant tuberculosis, namely bedaquiline (2012) and protonamid (2019). Extrapulmonary drug resistant-TB is a more severe and prevailing type of tuberculosis, posing a daunting diagnostic and therapeutic challenge [3]. With the prevalence of multidrug-resistant tuberculosis (MDR-TB) in previously treated cases of EPTB becoming alarming, it is important to develop strategies to curtail the prevalence of the infection, taking into consideration that an MDR-TB patient left untreated or inadequately treated will create another 10–15 new cases of MDR-TB in a year [4]. Lymph node tuberculosis encompasses one of the most common types of EPTB, representing approximately half of EPTB cases [1,5]. Additionally, the infection of lymph nodes can cause further EPTB complications as in the case of TB pericarditis (TBP), caused by the retrograde lymphatic spread of the bacilli from lymph nodes [6,7]. TBP is known to have a high burden in southern Africa due to the high prevalence of HIV and its contribution to TBP [7].

A limited number of studies have indicated that amongst the four standard anti-TB drugs, only isoniazid successfully permeates the pericardium at anti-tubercular concentrations. Rifampicin and ethambutol have poor permeation across the pericardium. Although pyrazinamide permeates across the pericardium, it lacks good anti-tubercular activity at a pH of 7.4, making TB pericarditis a difficult disease to treat with high mortality [8]. Studies have employed the use of nucleic acid amplification, corticosteroids, and colchicine therapy to improve diagnosis and treatment, prevent constrictive pericarditis, and decrease mortality, but to little avail [7,9]. Since the high bacillary burden is closely associated with mortality, for this reason, more research is needed to identify the optimal therapy, dose, duration, and delivery system to improve the treatment outcome of TB pericarditis.

Due to the difficulty in new drug development and adverse effects associated with the developed drugs, nanotechnology emerges as an important instrument in targeted delivery to extricate drugs with difficulty in use due to their poor solubility and high toxicity [10]. Bedaquiline (BDQ) is practically insoluble in aqueous media and has side effects as severe as QT prolongation. The incorporation of such drugs into nanoparticles (NPs) will improve drug selectivity and specificity to the required site of action whilst minimizing systemic side effects of the drug [11].

Chitosan is a biocompatible polymer that has mucoadhesive properties, is enzymatically biodegradable, and has a chemotactic action with concurrent epithelial permeability-enhancing properties [12]. Chitosan nanoparticles have previously been employed in the delivery of BDQ [13]. Apart from its general properties, it presents with various pharmacological actions, including antimicrobial, immunostimulant, and anti-inflammatory properties, that are advantageous for TB. It has been shown to have enhanced effects when used to transport poorly water-soluble drugs [14].

Chitosan oligosaccharide lactate (COS) is a water-soluble derivative of chitosan. This property allows for the synthesis of a nanosystem without having to use acidic media to solubilize the polymer, shorten the synthetic process, and accommodate acid-degrading co-polymers or drugs. COS has anti-inflammatory, antimicrobial, anti-oxidative, and tissue regenerative properties, as well as more favorable properties for certain biological interactions as compared to chitosan. The most prominent biological activity COS may have in TB would be its host-directed therapeutic activities. COS has immunomodulatory properties attained through its modulation of the nuclear factor kappa B (NF-κB) and mitogen-activated protein kinase (MAPK) pathways and its activation of the AMP-activated protein kinase (AMPK) pathway [15].

Chitosan has been reported to have the ability to open tight junctions and improve the paracellular diffusion of chitosan nanoparticles [16]. Cationic polymers such as chitosan are drug absorption enhancers through the reversible loosening of tight junctions [17]. Congruent with chitosan properties is the pericardial layer and its composite. The pericardium is made up of mesothelial cells, a type of epithelial cell with tight junctions, adherence junctions, gap junctions, and desmosomes [18]. For this reason, chitosan nanoparticles present a good strategy for the enhancement of drug permeation across the pericardium. On the other hand, permeation studies using chitosan oligosaccharide lactate (COS) have been conducted, and the results demonstrated enhanced permeation across the epithelial layer primarily through the transcellular pathway [19,20].

Macrophages are primary sites for TB infection, and due to the prominence of macrophages in the pericardial fluid and lymph nodes, they become sites of prolonged bacterial persistence [21]. Macrophages express various surface receptors, allowing for specific targets for drugs. The mannose receptor is one of them, allowing for the recognition of various molecules from the carbohydrate family for receptor-mediated endocytosis [22]. *M.tb* is further known to spread through macrophages in MDR (multidrug resistant)-XDR (extensively drug-resistant) TB, and for this reason, active targeting of macrophages may present a pathway in curtailing the disease.

Mannan oligosaccharide (MN) from *S. cerevisiae* is a polymer consisting of mannose, which has been employed as a targeting ligand for drug delivery unto CD206/mannose receptors on antigen-presenting cells such as macrophages [23]. In addition to its targeting abilities, it exhibits techno-functional properties, allowing it to act as a viscosity-enhancer and emulsion stabilizer [24]. It has been shown in earlier studies to have both anti-oxidative properties [25] and biological properties, including the inhibition of pathologic adherence, modulation of bacterial growth [26], and host-directed properties. Its properties improve host immune response by inducing IL10-producing, B-cell-regulated CD4^+^ T-cell polarization, ultimately resulting in a reduction in IFN-y (an anti-inflammatory response) and an increase in IL-4, which contributes to macrophage-induced tissue repair [23]. It additionally hinders the production of pro-inflammatory cytokines such as IL-1β, IL-6, and TNF-α, which correlates to the cytokines also found in TB pericarditis [27], suggestive of a prospective immunomodulatory effect in TB pericarditis. Mannan oligosaccharide additionally has epithelial protective properties by enhancing epithelial tight junctions [28] and, therefore, may have a supportive effect in the closing of tight junctions and maintaining epithelial integrity. With the permeation-enhancing properties of COS and the epithelial protective effect of MOS, their combinatory use may lead to a biopolymeric platform that might not disrupt the integrity of the pericardium whilst improving the permeation of the drug across it.

Various studies have investigated the incorporation of mannan onto the surface of nanoparticles for receptor-mediated targeting of macrophages [29]. Therefore, this study illustrates the novel incorporation of mannan onto chitosan oligosaccharide (COS) NPs as a ligand for the active targeting of nanosystems to surrounding lymph node components, e.g., Gata6^+^ macrophages, which are major components of the pericardial immune cell compartment [30]. The nanosystem is expected to concurrently enhance the permeation of BDQ across the pericardium. Given the localized nature of TB pericarditis, intrapericardial administration is proposed to enhance local drug exposure while minimizing systemic toxicity. The porcine pericardium is tested concurrently with the human pericardium to determine the similarity and interchangeable use of the tissue. The combination of BDQ, MN, and COS (with potential antibacterial activity) could yield a safer and more efficacious treatment for MDR-XDR tuberculosis. This nanosystem was thus synthesized and characterized for all relevant physicochemical and imaging modalities.

## 2. Results and Discussion

### 2.1. Assessment of BDQ-Loaded COS-MN NPs Regarding Particle Size, Zeta Potential, and Morphology

Chitosan NPs are prepared mainly by ionic gelation, whereby chitosan is dissolved in an acetic acid solution containing Tween 80, followed by the addition of TPP to allow cross-linking of the particles under stirring. Hydrophobic drugs are dissolved in an organic solvent such as dichloromethane and added dropwise into the chitosan solution before cross-linking with TPP [31]. In a previous study, bedaquiline-loaded chitosan nanoparticles have been synthesized with improved drug release compared to conventional NPs [32]. In this study, water-soluble COS and MN in the presence of Tween 80, without acetic acid, were cross-linked with TPP for the encapsulation of BDQ.

**Figure 1:**
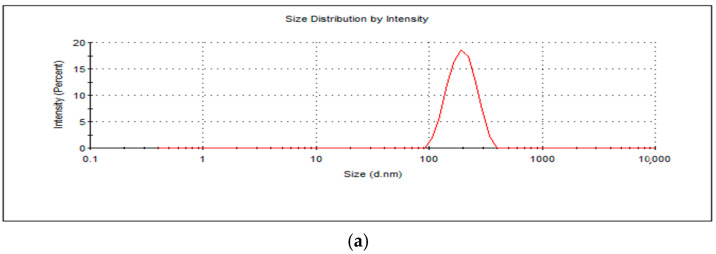

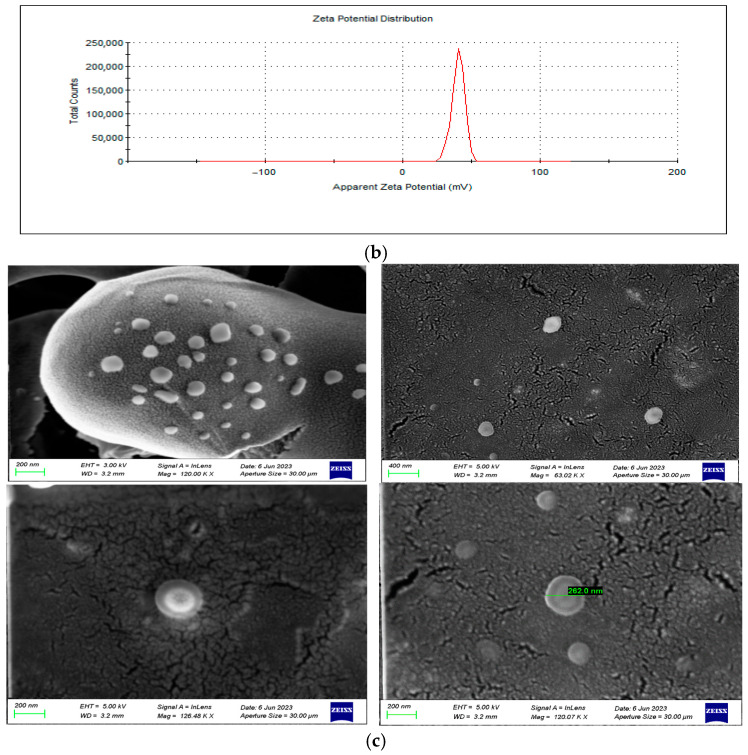
(**a**) A graphical representation of BDQ-loaded, mannan-conjugated COS NPs’ average hydrodynamic size, (**b**) surface potential, and (**c**) smooth electron microscopy (SEM) images at magnification (top left (120.00 kx, EHT = 3.00 kV); top right (63.02 kx, EH 5.00 kV); bottom left (126.48 kx, EH 5.00 kV); bottom right (120.07 kx, EHT = 5.00 kV).

The average hydrodynamic size and zeta potential were measured as 192.4 nm (Figure 1a) and 40.5 mV (Figure 1b), respectively, and the overall morphology of the nanosystem was found to be roughly spherically shaped as per smooth electron microscopy imaging (SEM) (Figure 1c). Due to the 5 kV EHT applied on the nanosystem in imaging as compared to 3 kV EHT, various layers are observed on the nanosystem. The outermost layer of the nanosystem melted, which could be attributed to mannan, which is heat sensitive, confirming the presence of mannan on the surface. The size was thus in the desired 100–200 nm size range for macrophage internalization [33]. Furthermore, the cationic potential and presence of mannan will allow for high uptake of the nanoparticles by the macrophage’s negatively charged sialic acid and mannose receptors, respectively [28,34]. The nanoparticles’ Polydispersity Index (PDI) was recorded as 0.199, indicating colloidal stability and minimal aggregation in dispersed aqueous media. The PDI and the zeta potential concurrently demonstrated viability as a systemic nano-drug delivery system. The high surface charge, while beneficial for cellular uptake, may promote nonspecific interactions with biological components and warrants further studies, including serum protein binding and cytotoxicity assays.

Although preliminary storage of lyophilized nanoparticles has shown no macroscopic or re-dispersion issues over 3 months, formal stability studies are necessary to determine their shelf life, reconstitution behavior, and long-term physicochemical stability.

### 2.2. Assessment of Functional Transformation of the BDQ-Loaded COS-MN NPs

**Figure 2:**
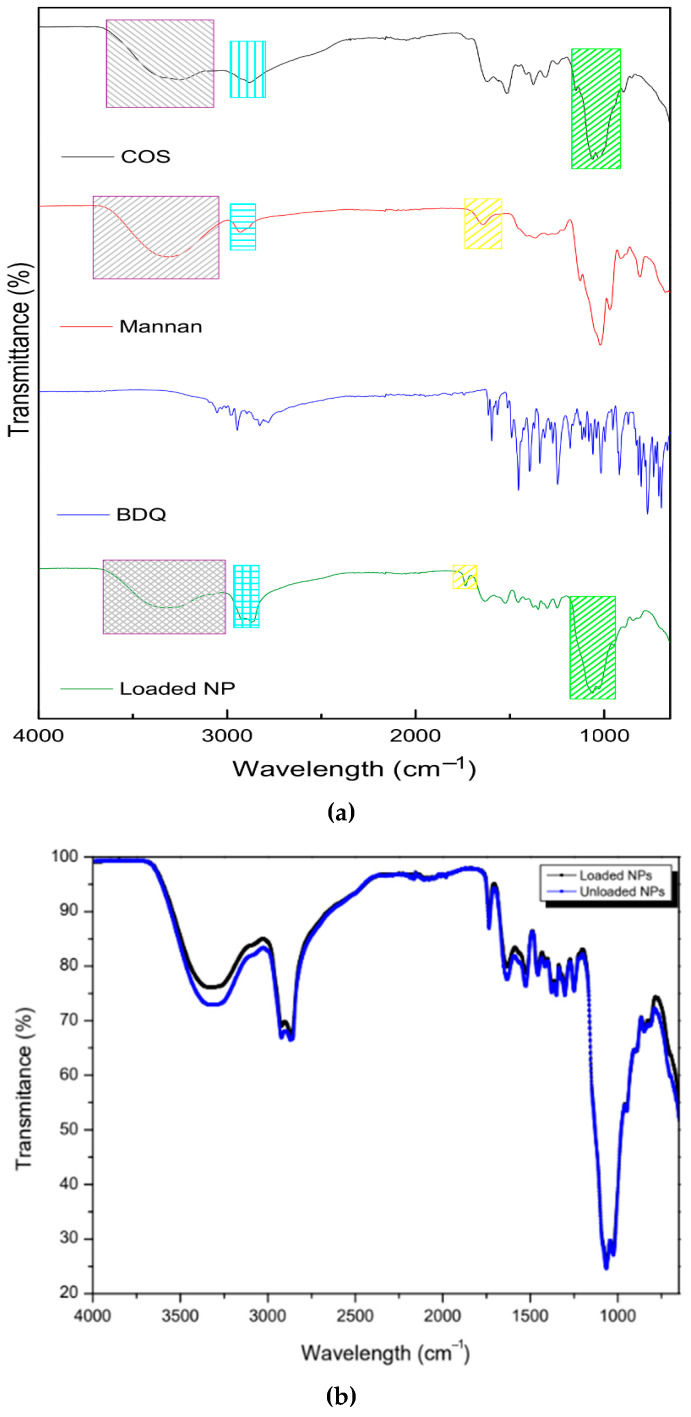
FTIR images showing (**a**) the pristine mannan, chitosan oligosaccharide lactate, bedaquiline, loaded NPs, and (**b**) unloaded NPs vs. drug-loaded NPs.

The FTIR spectra of COS, MN, BDQ, and the drug-loaded nanoparticles are depicted in Figure 2. The pristine COS exhibited characteristic broad absorption peaks at 3248 cm^−1^, which is attributed to OH stretch. The CH- stretch appeared at 2883 cm^−1^, whilst the C=O stretch from the lactate derivative and amide (II) bending appeared at 1618 cm^−1^ and 1586 cm^−1^, respectively. The 1376 cm^−1^ absorption peak indicates C-C bending. The intense absorption peaks at 1062 cm^−1^ and 1028 cm^−1^ are attributed to the asymmetric C-O-C bridge and skeletal vibrations characteristic of its saccharide structure [35]. These peaks were in correspondence with the observed peaks on the drug-loaded nanosystem: 3338 cm^−1^ (O-H stretch), 2870 cm^−1^ (C-H stretch), 1632 cm^−1^ and 1523 cm^−1^ (C=O stretch and NH_2_ bending), and 1063 cm^−1^ and 1028 cm^−1^ (asymmetric C-O-C bridge). The shift in the amine and amide peaks indicates cross-linking between COS and sTPP [36].

The pristine mannan exhibited a broad absorption peak at 3320 cm^−1^, indicative of the OH stretch. The peak at 2929 cm^−1^ represents the symmetric and asymmetric CH-CH-stretching vibrations, which are characteristic of polysaccharides [37]. The pyranose ring (C-O-C stretching) of mannan is suggested by the 1021 cm^−1^ peak. Absorption peaks at 913 cm^−1^ and 811 cm^−1^, which are used to access purity, are also characteristic of mannan, further indicative of an α-glycosidic linkage in the pyranose of mannan [38]. The peaks were in accordance with the observed peaks on the drug-loaded nanosystem—3338 cm^−1^ (O-H stretch) and 2870 cm^−1^ (C-H stretch)—with a higher absorbance due to the co-polymeric complex with COS. The peak at 1643 cm^−1^ on mannan (C=O stretching) shifted and became sharper on the loaded nanoparticle to 1735 cm^−1^, indicative of the co-polymeric complexation. Oligosaccharide chains are short and contain 2–10 sugar units [39]. This complexation may occur by a ring opening and proton transfer from the O–H group of either polymer at the beginning or end of the chain to the O within the pyranose ring of either polymer, thereby forming a carbonyl group (C=O) [40]. The pristine bedaquiline spectra showed characteristic peaks of ether (1180 cm^−1^ and 1081 cm^−1^) and aromatic (3054 cm^−1^, 3028 cm^−1^, 2975 cm^−1^, 2946 cm^−1^, 1614 cm^−1^, 1596 cm^−1^, 1455 cm^−1^, and 1250 cm^−1^) functional groups [41]. In the drug-loaded nanoparticles, most peaks disappeared, confirming the encapsulation of the nanosystem, with only faint absorbance at 1455 cm^−1^ and 1250 cm^−1^, showing the presence of bedaquiline in the nanosystem. The unloaded nanosystem displayed the same peaks as the loaded nanosystem. The absorbance at 3248 cm^−1^, which is attributed to OH stretch, was observed to a greater extent in the unloaded nanosystem. This may be due to the interaction of chitosan with bedaquiline, covering up the bond as compared to the unloaded nanosystem.

### 2.3. Determination of Powder Diffraction of BDQ-Loaded COS-MN NPs in Comparison to Individual Compounds

**Figure 3:**
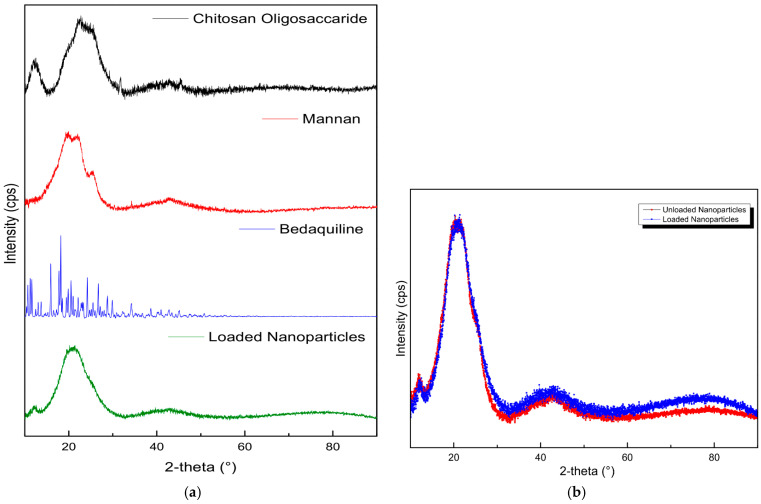
X-ray diffraction images of (**a**) the pristine mannan, chitosan oligosaccharide lactate, bedaquiline, loaded NPs, and (**b**) unloaded NPs vs. drug-loaded NPs.

The diffraction patterns in Figure 3 show the crystalline nature of pristine BDQ, COS, and MN and how they interact to form a nanoparticle. BDQ was observed to be highly crystalline, with significant peaks at 2θ-10.5°, 11.5°, 16°, 18°, 20.5°, 24°, and 26.5°. Pristine COS exhibited typical saccharide amorphous peaks at 12° and a semicrystalline peak at 23° [42]. Mannan showed a semi-crystalline peak at 20°, corresponding in intensity with the 21.5° peak on MN-COS BDQ-loaded nanoparticles. The loaded NPs’ peak was a smoother semi-crystalline peak, due to a slight phase change in the cross-linking of mannan with COS. The absence of BDQ crystalline peaks on the nanoparticles confirms the complete encapsulation of the drug in the co-polymeric matrix.

### 2.4. Thermo-Behavioral Analysis of BDQ-Loaded COS-MN NPs

**Figure 4:**
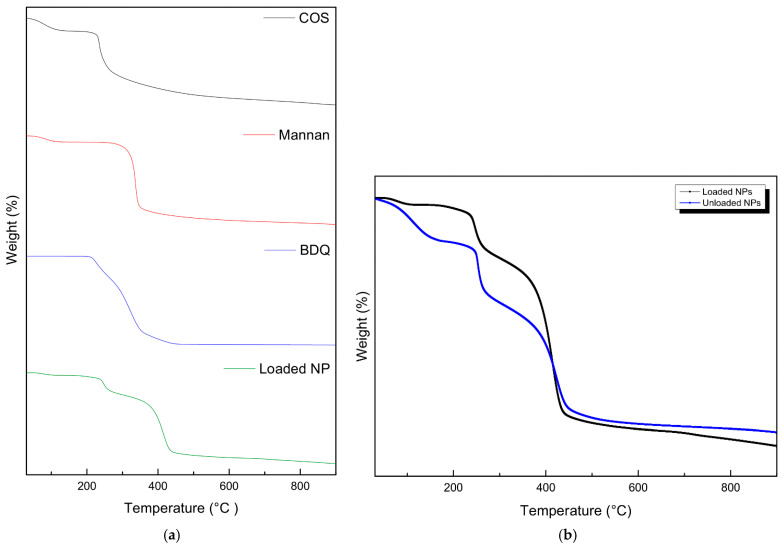
Thermogravimetric analysis of (**a**) the pristine mannan, chitosan oligosaccharide lactate, bedaquiline, loaded NPs, and (**b**) unloaded NPs vs. drug-loaded NPs.

As in Figure 4, the pristine COS, mannan, and drug-loaded NPs showed a conserved weight loss below 100 °C due to dehydration. At 230 °C, 310 °C, and 220 °C, a decline is noticed for COS, mannan, and BDQ, respectively, indicating significant degradation of the components. The loaded NPs showed a two-step degradation at 240 °C and 380 °C, corresponding to the loss of COS and mannan, respectively. The shift in the exothermic peaks observed in the nanoparticles infers partial cross-linking between sTPP and COS and interpolymer complexation between COS and mannan, resulting in a transition in thermal properties, indicated by an increase in the degradation temperature of the respective components. The unloaded nanoparticles showed an initial weight loss between 100 and 200 °C like that observed in COS. This degradation is not as significant in the loaded nanoparticles, possibly suggesting the presence of bedaquiline binding to COS, thus preventing its degradation.

### 2.5. Syringeability and Rheological Properties of BDQ-Loaded MN-COS NPs

**Figure 5:**
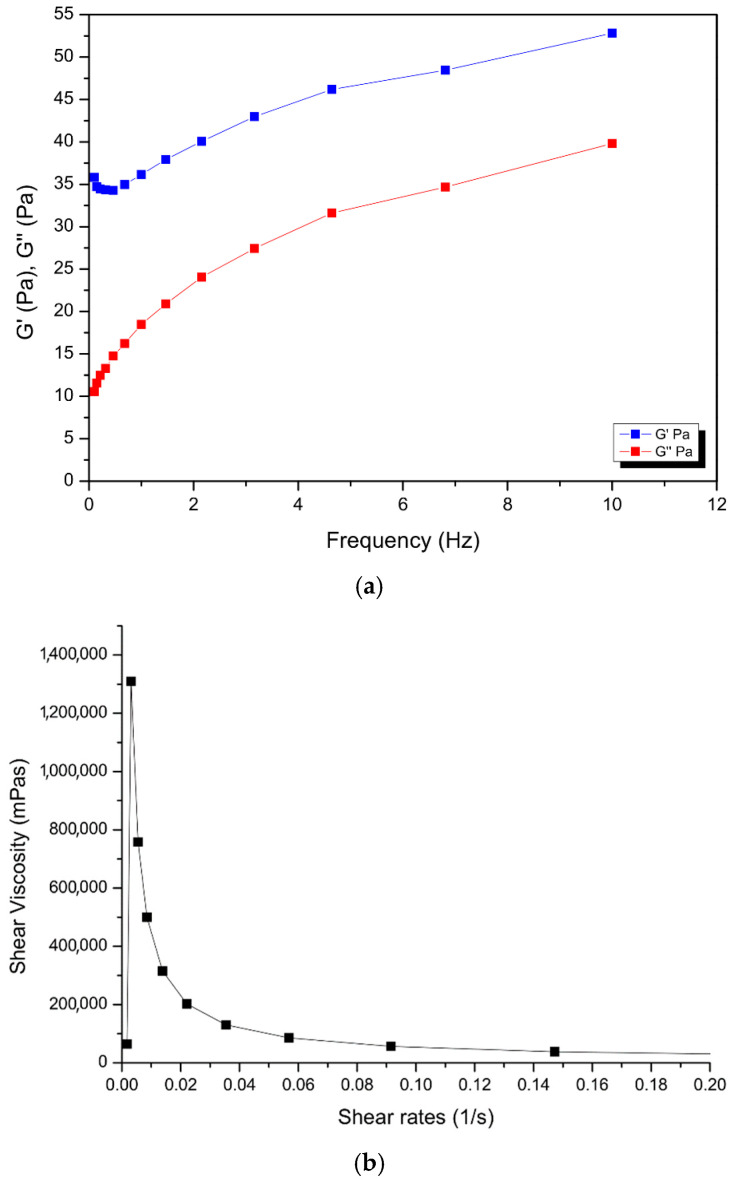
Rheological images showing (**a**) frequency sweep and (**b**) viscosity sweep of the nanogel.

The nanosystem showed gel-like behavior, whereby G′ > G″ as can be observed in Figure 5 below. The shear viscosity of COS-MN-NPs at 37 °C with varying shear rates from 0 to 2000 s^−1^ suggests a shear thinning property in the gel as the viscosity decreases with an increasing shear rate. The viscoelastic nature (elastic (G′) and viscous (G″) moduli) of the nanosystem was also evaluated. Higher values of the G′ modulus with a higher frequency indicated better gel strength at 37 °C [43]. The gel solution is found to be partially diluted as the difference in G′ and viscous G″ get closer to each other at higher frequency values. The viscoelastic modulus ranged from 10.56 to 52.82 Pa for the gel across the frequency range, showing applicable use in the pericardial space, without significantly affecting the pressure, since the effective pericardial pressure is between 0 and 133.32 Pa [44]. The syringeability test was performed in triplicate under a flow rate of Q = 0.1 mL.s^−1^. The injection force was found to be 11.94 N, well below the maximum force of 79.8 N that can be generated and applied on a syringe by humans (males: 95.4 N; females: 64.1 N) [45].

### 2.6. Evaluation of Mannan Content, BDQ-Loading Capacity, and Drug Entrapment Efficacy of BDQ-Loaded COS-MN NPs

The phenol–sulfuric acid method works by sulfuric acid digestion and dehydration of the polysaccharide mannan to monosaccharide mannose (a hexose sugar), which further reacts with phenol to produce hydroxymethyl furfural, a yellowish-brown colored mixture [46]. In this study, the amount of mannan attached to the nanosystem was calculated to be 94.86%. The BDQ-loading capacity was calculated to be 0.6%, and the BDQ entrapment efficiency was 98.76% using RP-HPLC.

### 2.7. Evaluation of In Vitro Release Kinetics of BDQ from the BDQ-Loaded COS-MN NPs

**Figure 6:**
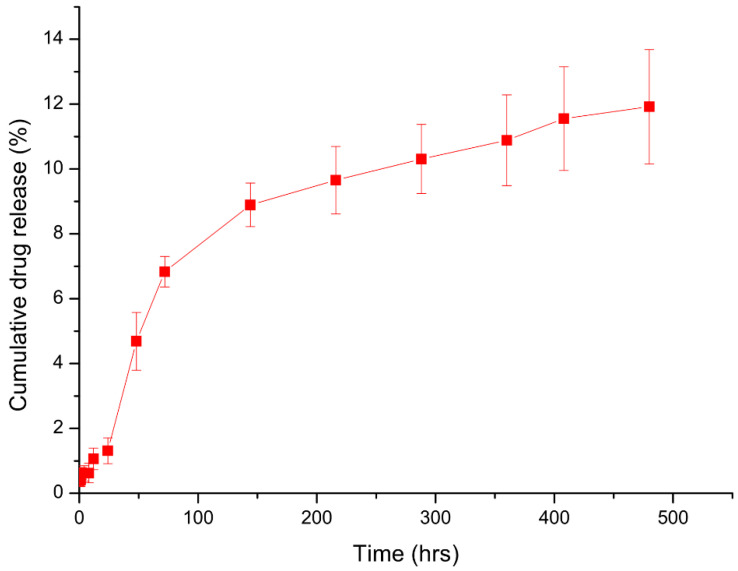
In vitro bedaquiline-loaded nanoparticle drug release at pH 7.4 over 20 days (n = 3). Error bars = SEM.

The release kinetics of BDQ were evaluated at pH 7.4 in 0.1 M PBS containing 0.2% SLS to mimic the pH of 7.34 of the pericardial fluid of pericardial tuberculosis [8]. The BDQ release at pH 7.4 reached a steady state from day 3 of release, with an average of 11.92% of the drug released after 20 days, as depicted in Figure 6. Since bedaquiline is effective at very low concentrations, with as low a MIC of 0.0039–0.25 µg.mL^−1^ [47], the nanosystem demonstrates potential for sustained drug delivery at therapeutically relevant levels. These findings suggest the possibility of a long-acting formulation suitable for single-dose administration, warranting further translational investigation.

### 2.8. Evaluation of Ex Vivo Diffusion Study of the BDQ-Loaded MN-COS NPs Across Porcine and Human Pericardia

**Figure 7:**
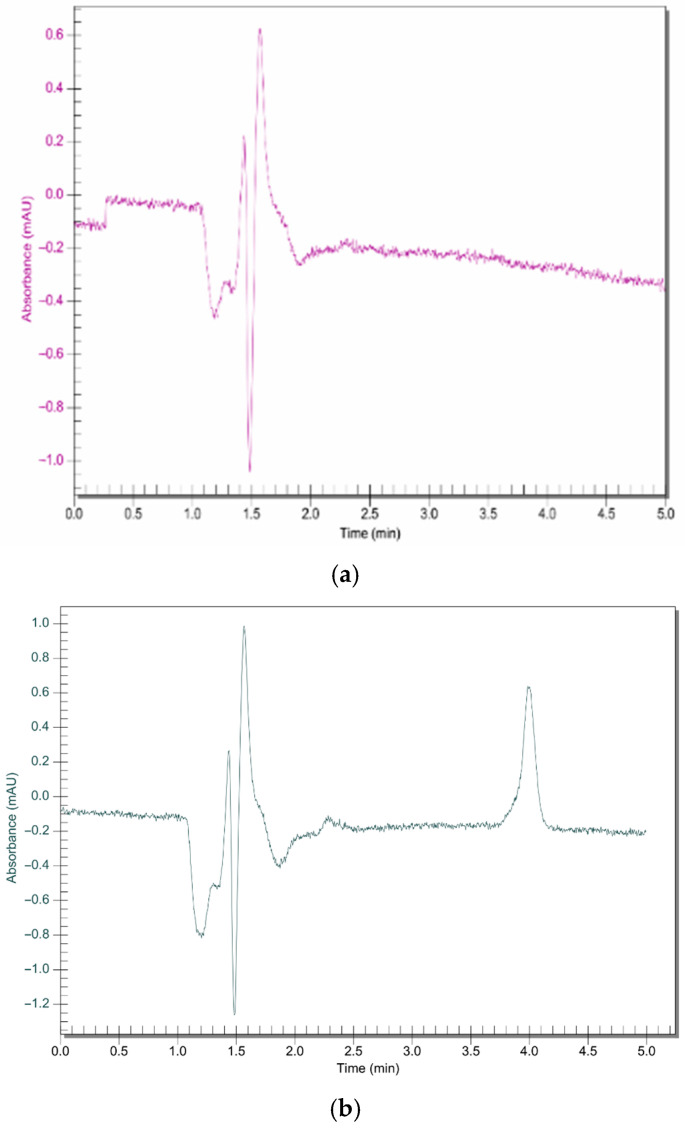
RP C18 HPLC chromatograms for (**a**) unloaded nanosystem and (**b**) bedaquiline-loaded nanosystem (Rt = 4.01; concentration= 0.2 ug/mL). Run time of 5 min using a mobile phase of 95:5 (organic phase (containing methanol 85:15 acetonitrile)/phosphate buffer) at a flow rate of 1 mL.min^−1^ and a detection wavelength of 275 nm.

**Figure 8:**
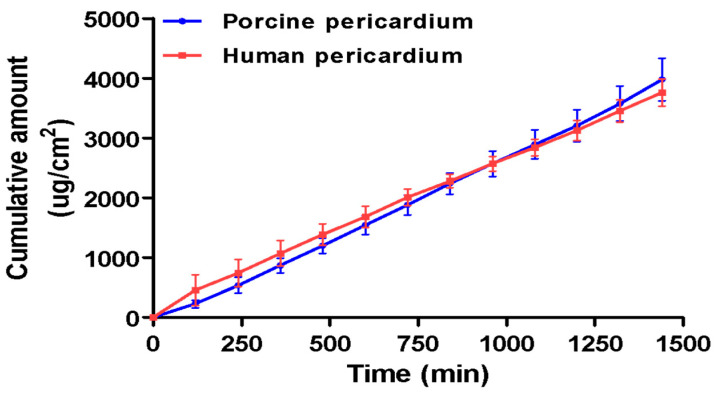
Cumulative amount vs. time graph of bedaquiline diffusion from the bedaquiline-loaded nanosystem across ex vivo porcine and human pericardia (n = 14). Error bars = SEM. Due to the insolubility of bedaquiline in aqueous media, a bedaquiline aqueous solution was impossible to test across the tissue, therefore necessitating the incorporation of the drug into a nanosystem.

The RP-HPLC method successfully quantified the amount of bedaquiline that is diffused across the pericardium without interference from the nanoparticle components (Figure 7). Human and porcine pericardia displayed comparable results in their average cumulative permeation graphs (Figure 8). Steady-state flux values were reached as quickly as 2 h across both human and porcine pericardia. The steady-state fluxes were calculated to be 2.889 and 2.346 µg.cm^−2^.min^−1^ for porcine and human pericardia, respectively; the apparent permeability coefficients were calculated to be 2.66 × 10^−4^ cm.min^−1^ and 2.16 × 10^−4^ cm.min^−1^ for porcine and human pericardia, respectively. The lag phases were 52.72 min and 0 min for porcine and human pericardia, respectively. Bedaquiline diffusion across porcine pericardium was found to be slightly higher compared to human pericardium.

The results obtained indicate that the porcine pericardium represents a good model for human pericardium for ex vivo diffusion studies, as there were no statistically significant differences (*p* > 0.05) found between the steady-state flux rates and apparent permeability coefficients of bedaquiline between porcine and human pericardial tissues. Both specimens showed a linear diffusion of the bedaquiline nanoparticle, with an instant to speedy steady-state establishment and constant cumulative drug permeating over time. With poor penetration of standard anti-tuberculosis treatment (ATT) across the pericardium, other interventions needed to be identified to treat the second most lethal version of extrapulmonary tuberculosis, tuberculosis pericarditis. Intrapericardial injections during pericardial effusion are an established route in clinical settings, which could allow for direct delivery of the nanoparticles to the infected pericardium, potentially enhancing therapeutic concentration while avoiding systemic exposure. The results obtained in this study therefore clearly indicate prospects of the nanosystem to allow for the permeation of bedaquiline at an effective concentration consistently. The slow, sustained permeation profile delivery and long half-life of bedaquiline suggests the potential for infrequent dosing, possibly once every two weeks. Localized delivery to the pericardium may reduce systemic exposure and thereby mitigate dose-dependent side effects such as QT interval prolongation, which remains a major limitation of systemic BDQ therapy.

Bedaquiline is a very lipophilic molecule with a molecular weight of 555.50 g.mol^−1^ and a very high log P value of 7.74 (PubChem). No ex vivo permeation studies have been performed on pericardial tissue to determine its correlation of log P to permeation across the tissue to date, but studies on other tissues in the body have reported that the permeability coefficient and log P demonstrate a proportional relationship [48]; therefore, a compound with a high log P (more lipophilic molecule) value is expected to permeate the pericardium better.

The improved permeation and drug release of bedaquiline ex vivo as compared to in vitro dissolution studies may be attributed to the presence of biofactors in snap-frozen tissues [38]. Chitosan/COS is prone to lysosomal biodegradation through hydrolysis [12,49]. Lysosomes are present in all human cells, so the improved permeation and release of bedaquiline, as evident in the results, may be due to the lysosomal factor on the nanosystem after transcellular permeation, since chitosan permeates through paracellular/transcellular pathways [50]. This may suggest a controlled drug-release polymeric nanosystem in which drug release occurs by degradation/erosion of the matrix rather than diffusion [49,51].

### 2.9. Tissue Integrity Study

The integrity of the porcine and human pericardial tissues over 24 h of exposure to the bedaquiline nanosystem was evaluated using tissue resistance measurements. The transepithelial electrical resistance change across the pericardial tissue before and after the diffusion study (n = 3) showed no statistically significant change, with a mean percentage difference of 4.43 ± 0.49% and 2.78 ± 1.14% (*p* > 0.05) for porcine and human pericardia, respectively (Table 1). This implies that the tissue integrity was not affected by the nanosystem or the collecting media throughout the experiment.

**Figure 9:**
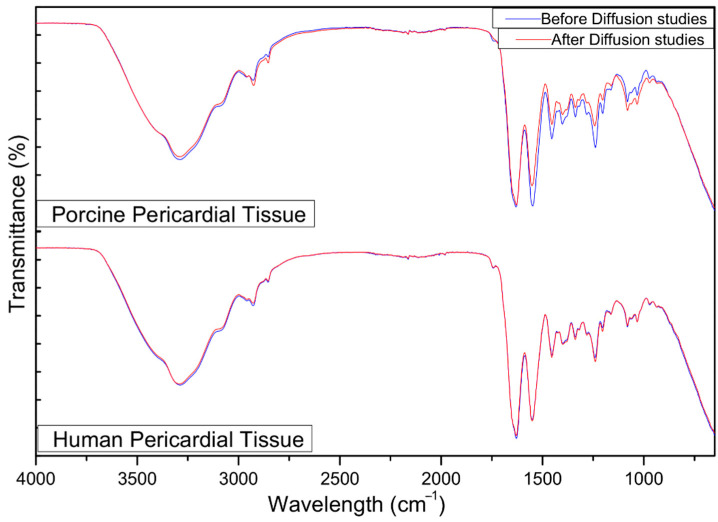
FTIR images of the porcine and human pericardia before and after 24 h of the ex vivo diffusion study.

Pericardium contains mainly Type-1 collagen, glycoproteins, and glycosaminoglycans [52]. This is concurrent with the characteristic absorption peaks between 3000 cm^−1^ and 2800 cm^−1^ showing lipid hydrocarbon absorption. The peak at 1630 cm^−1^ is attributed to amide I-C=O stretching of the backbone of the proteins. Furthermore, the peak at 1550 cm^−1^ indicates an amide II bond, showing N-H bending coupled to C-N stretching. The 1454 cm^−1^ peak shows asymmetric and symmetric C-H bending from CH_2_ and CH_3_ on proteins. The 1400 cm^−1^ peak is indicative of C=O vibrations of COO^−^ from free amino acids and polypeptides in the pericardial structure. Amide III from proteins is characterized by the 1338 cm^−1^ peak. The 1033 cm^−1^ peak indicates C-O vibrations from polysaccharides in glycoproteins and glycosaminoglycans [53]. There was immutability in the transmittance of the bonds on the mesothelial pericardial tissue across the range of 4000–650 cm^−1^ over 20 scans before and after the diffusion study (Figure 9). There were no statistically significant differences (*p* > 0.05) before and after the diffusion study, suggesting that tissue integrity was not compromised by the nanoparticle or collecting media.

Chitosan oligosaccharide (COS) has favorable biodegradation via endogenous lysozymes, with minimal systemic toxicity. Mannan conjugates are known to interact with antigen-presenting cells (APCs) through mannose receptors and, in combination with COS, may induce a synergistic immunostimulatory response. This host-directed effect could be beneficial in the treatment of TB pericarditis by modulating macrophage and APC activities. For pericardial applications, however, immune activation and phagocytic clearance may influence both therapeutic efficacy and systemic tolerability. While both mannan and COS are generally considered biocompatible, their immunogenicity and in vivo clearance profiles warrant further investigation. These aspects will be explored in future in vitro and in vivo studies.

Although no cytotoxicity or biocompatibility studies were included in this initial investigation, we acknowledge the importance of such data in assessing translational potential. Future studies will investigate the cytotoxic and inflammatory responses of the nanosystem in vitro using relevant human cardiac and pericardial cell lines to ensure safety and compatibility.

## 3. Materials and Methods

### 3.1. Materials

The materials employed in this study were of the highest purity grade and were purchased from Sigma-Aldrich (Pty) Ltd. (St. Louis, MO, USA). They included chitosan oligosaccharide lactate (COS) (Mw 5 kDa), mannan oligosaccharide (MN) from *Saccharomyces cerevisiae*, Tween 80, dichloromethane (DCM), sodium tripolyphosphate (sTPP), bedaquiline (BDQ), ethanol, phenol, sulfuric acid (H_2_SO_4_), methanol (HPLC grade), acetonitrile (HPLC grade), potassium dihydrogen orthophosphate, triethylamine, orthophosphoric acid, phosphate-buffered saline (PBS) tablets, and sodium lauryl sulphate (SLS).

### 3.2. Synthesis of the BDQ-Loaded COS-MN NPs

**Figure 10:**
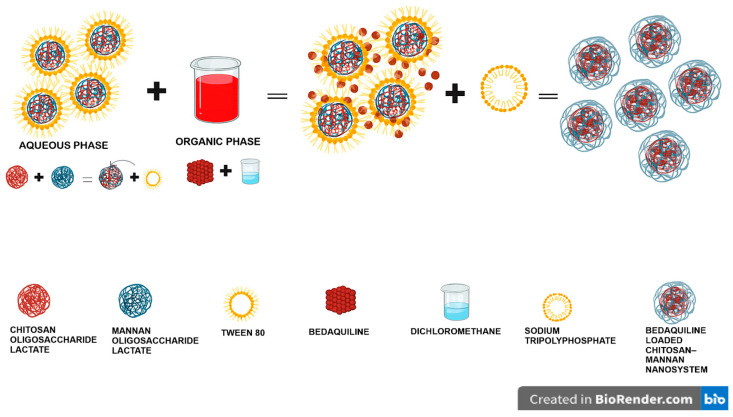
A schematic diagram overview showing how the various components combine in the preparation of the COS-MN-BDQ NPs.

The bedaquiline-loaded mannan–COS NPs were prepared by a one-step ionic gelation probe sonication method, as illustrated in Figure 10. COS (200 mg) was added into 10 mL of distilled water, followed by the addition of mannan (50 mg) into the solution and stirring for 30 min. Tween 80 (200 μL) was added dropwise into the solution until a homogenous solution was obtained. Bedaquiline (5 mg) was dissolved in 5 mL of DCM, and the aqueous solution was added to the organic solution, followed by stirring at 1500 rpm for 20 min. sTPP (7.5 mg) was dissolved in 5 mL of DW and filtered through a 2.2 µm filter. The sTPP was added dropwise into the prepared bedaquiline solution at a 0.2 mL.min^−1^ drop rate, after which the solution was stirred for 24 h. The resultant solution was then homogenized at 5000 rpm for 10 min. The suspension obtained was sonicated (CV33 VibraCell, Sonics and Materials, Inc., Danbury, CT, USA) for 2 min at 4.8 million Joules and 40% amplitude. The suspension was frozen at −80 °C for 48 h and then subsequently lyophilized for 12 h (FreeZone-50C Benchtop Freeze Dryer, Labconco, Kansas City, MO, USA).

### 3.3. Physicochemical and Physicomechanical Characterizations

#### 3.3.1. Determination of Particle Size, Zeta Potential, and Morphology

A nano ZetaSizer instrument (NanoZS, Malvern Panalytical, Malvern, UK) was used to obtain the average hydrodynamic size of the nanoparticles and the surface zeta potential of the nanoparticles by dynamic light scattering and electrophoretic light scattering, respectively. The suspension (0.1 mL) was diluted into 10 mL of distilled water. The resultant samples (1 mL) were transferred into disposable cuvettes to determine the hydrodynamic size and Polydispersity Index, while 0.8 mL was transferred into zeta potential disposable folded capillary cells (DTS 1070, Malvern Panalytical) for surface charge analysis at 25 °C.

#### 3.3.2. Fourier Transform Infrared (FTIR) Spectroscopy: Physicochemical and Physicomechanical Characterizations

FTIR spectroscopy (Spectrum 100, PerkinElmer Inc., Waltham, MA, USA), with an Attenuated Total Reflectance (ATR) accessory, was used for comparative analysis of nanoparticles and individual components to identify the difference in the vibrational transition of the chemical bonds in the molecule. The bonds present will confirm the encapsulation of the drug in the nanosystem. Samples of individual BDQ, mannan, COS, and BDQ-loaded COS-MN nanoparticles were analyzed at 110 psi pressure in a range of 4000–650 cm^−1^ over 20 scans to obtain relevant spectra.

#### 3.3.3. X-Ray Diffraction (XRD) Analysis

The degree of crystalline transition of pristine BDQ, MN, COS, and BDQ-loaded COS-MN nanoparticles was analyzed employing X-ray diffractometry (XRD) (Rigaku MiniFlex, Rigaku Inc., Tokyo, Japan). The dry samples of BDQ, MN, COS, and BDQ-loaded COS-MN nanoparticles were mounted onto aluminum sample holders and measured at a current of 40 kV and 15 mA CuKα radiation. The scanning measurement was in the 2θ range of 10–90°, with a scan rate of 10°.min^−1^.

#### 3.3.4. Thermogravimetric Analysis (TGA)

The extent of thermal degradation of pristine BDQ, MN, COS, and BDQ-loaded COS-MN nanoparticles was evaluated using a thermogravimetric analyzer (PerkinElmer, TGA 4000, Llantrisant, Wales, UK). The samples were heated up from 30 °C to 900 °C at a 10 °C.min^−1^ heating rate under a constant and continuous 19.8 mL.min^−1^ nitrogen flow. The thermograms were extrapolated as the percentage weight dependent on temperature.

#### 3.3.5. Gelling Properties and Texture Analysis of BDQ-Loaded COS-MN NPs

Rheological measurements, such as the shear viscosity at varying shear rates and frequency sweep, for the hydrogel base were performed using the Haake Mars (II) Modular Advanced Rheometer system (Thermoscientific, Waltham, MA, USA). This was undertaken using a cone plate geometry (Rotor C35/1, D = mm, 1° Titan) at a gap distance of 0.052 mm. The COS-MN-BDQ-NPs base shear viscosity was determined at varying shear rates at 37 °C. Oscillatory frequency sweeps for COS-MN-BDQ-NPs were performed at a constant shear stress of 1 Pa in the frequency range from 10 to 0.10 Hz, at 37 °C. The syringeability of the concentrated COS-MN-NPs solution was studied using a texture analyzer (TAXT2, Stable Microsystems, Godalming, UK) in compression mode, whereby the force required to be exerted on the plunger of the syringe to inject the gel was evaluated. The COS-MN-BDQ-NPs gel (1 mL) was drawn into 3 mL syringes and equipped with a 21 G needle. A force transducer connected to the texture analyzer was employed to measure the force required to push the plunger of the syringe. The displacement rate was 10 mm/s, which corresponds to a flow rate of 1 mL.min^−1^.

#### 3.3.6. Evaluation of Mannan Loading Content, BDQ-Loading Capacity, and Drug Entrapment Efficacy of BDQ-Loaded COS-MN NPs

Conjugated mannan was investigated using a colorimetric assay by a phenol–sulfuric acid method. Using a Nanophotometer UV/Vis spectrophotometer NP80 (Implen, Munchen, Germany), the total sugar was spectrophotometrically measured at 490 nm, the wavelength at which hexoses and their methylated derivatives show maximum absorption [54,55]. Unloaded nanoparticles were synthesized following the same method without adding bedaquiline. The supernatant and washing solution for both a drug-loaded and drug-free system were collected and used to quantify unconjugated mannan by a phenol–sulfuric acid method. Briefly, 1 mL of 5% phenol was added into 2 mL of supernatant solution, followed by a rapid addition of 5 mL of 96% H_2_SO_4_ and heating at 90 °C for 5 min, followed by 20 min of cooling. The drug-free system supernatant was used as the blank. The absorbance was then measured and extrapolated from a standard curve of 1 µg.mL^−1^, 5 µg.mL^−1^, 10 µg.mL^−1^, 15 µg.mL^−1^, and 20 µg.mL^−1^ of mannan. The conjugated mannan was calculated using the following Equation (1).

(1) $$\frac{\mathrm{Total}\;\mathrm{mannan}\;\mathrm{added}-\mathrm{Unloaded}\;\mathrm{mannan}}{\mathrm{Total}\;\mathrm{mannan}\;\mathrm{added}}\;\times \;100\%$$

Synthesized nanoparticle entrapment efficiency and drug loading were determined by centrifugal washing (n = 3) using 0.2% SLS in water, conducted in triplicate, at 12,000 rpm 25 °C for 10 min using an Ultracel^®^ Regenerated Cellulose membrane (12 kDa MWCO), with 15 mL sample volume (Sigma-Aldrich (Pty Ltd. (St. Louis, MO, USA)). The unreacted supernatant was collected and diluted with methanol/acetonitrile (85:15) and run in triplicate on the HPLC. The obtained results were inserted into Equations (2) and (3) below and used to calculate the percentage.

(2) $$\mathrm{Entrapment}\;\mathrm{efficiency}=\frac{\left(\mathrm{Total}\;\mathrm{amount}\;\mathrm{of}\;\mathrm{drug}-\mathrm{Unloaded}\;\mathrm{drug}\;\mathrm{in}\;\mathrm{supernatant}\right)}{\mathrm{Total}\;\mathrm{amount}\;\mathrm{of}\;\mathrm{drug}}\;\times \;100\%$$

(3) $$\mathrm{Drug}\;\mathrm{Loading}=\frac{\left(\mathrm{Total}\;\mathrm{amount}\;\mathrm{of}\;\mathrm{drug}-\mathrm{Unloaded}\;\mathrm{drug}\;\mathrm{in}\;\mathrm{supernatant}\right)}{\mathrm{Total}\;\mathrm{weight}\;\mathrm{of}\;\mathrm{formulation}}\;\times \;100\%\;$$

#### 3.3.7. In Vitro Drug Release Studies

The BDQ release profile from the BDQ-loaded MN-COS-NPs was examined at pH 7.4 (pericardial space pH) by loading 105 mg.mL^−1^ NPs dispersed in 0.1 M PBS into semipermeable dialysis membrane tubing (SnakeSkin™, 3500 MWCO) (Sigma Aldrich, St. Louis, MO, USA). The dialysis membrane tubing was then submerged into 40 mL of dissolution medium consisting of 40 mL of PBS containing 0.2% (*w*/*v*) SLS. SLS was used to ensure the solubility of BDQ in dissolution media due to its hydrophobic nature. The dissolution study was performed in a thermostatically stable orbital shaking incubator (Yihder LM-530, Yihder Co., Ltd., Taipei, Taiwan) at 37 °C. At different time intervals (t = 0.5, 1, 2, 4, 8, 12, 24, 48, 72, and 144 h), 1 mL of each sample was withdrawn with concurrent replenishing of an equivalent amount of dissolution medium to maintain sink conditions. The released BDQ was quantified using an HPLC method using a Phenomenex (Torrance, CA, USA) Kinetex C18 Reverse-Phase Liquid Chromatography Column with dimensions of 150 × 4.6 mm and a particle size of 5 µm. The mobile phase comprised an organic phase of (a) methanol/acetonitrile (85:15 *v*/*v*) and an aqueous phase (B) made of triethylamine (10 mL in 1000 mL) and potassium phosphate buffer (20 mg in 1000 mL) adjusted with orthophosphoric acid to a pH of 7.4. The solvents were pumped across the HPLC at a ratio of 95:5 *v*/*v* (A: B). The flow rate was set at 1.0 mL.min^−1^ with a run time of 5 min. The detection wavelength on the UV/Vis detector was set at 275 nm. The method was linear within the range of 1–50 μg.mL^−1^.

To ensure accurate quantification of bedaquiline (BDQ) during the in vitro release studies, the HPLC method was validated with a limit of detection (LOD) of 0.05 µg/mL and a limit of quantification (LOQ) of 0.15 µg/mL, determined using a signal-to-noise ratio method. Release profiles were first evaluated using two different amounts of the nanosystem (2-fold difference in input drug content) to assess reproducibility and confirm that sink conditions were maintained. The maximum cumulative BDQ concentrations observed after 20 days were 0.64 µg/mL and 1.47 µg/mL for the lower and higher nanosystem doses, respectively. These concentrations fall within or just below the estimated solubility range of BDQ in PBS containing 0.2% SLS (approximately 5 µg/mL), confirming that sink conditions were upheld throughout the experiment. The use of varying nanosystem doses, combined with a sensitive and validated analytical method, enhances the reliability of the release kinetics data and supports that the results were not confounded by solubility limitations or analytical constraints.

### 3.4. Collection and Treatment of Porcine and Human Pericardial Tissues

#### 3.4.1. Porcine Pericardial Tissue

Porcine pericardial tissue was collected from animals euthanized for other purposes at the University of the Witwatersrand Central Animal Unit. For this study, an ethics waiver for the use of porcine tissue was obtained from the University of the Witwatersrand Animal Research Ethics Committee (waiver number: AREC-101210-002).

The pericardial tissue was immediately placed in phosphate-buffered saline (PBS, pH 7.4) and, within an hour, sectioned into strips, placed into cryovials, snap frozen, and stored in liquid nitrogen (−195 °C).

#### 3.4.2. Human Pericardial Tissue

Healthy pericardial specimens were collected from patients (>18 years of age) who underwent routine cardiac surgery at the Kuils River Netcare Hospital. Tissues collected were designated to be disposed of as medical waste. Ethics approval was obtained for the use of human pericardial tissue from the University of the Witwatersrand Human Research Ethics Committee (approval number: M230545). The pericardial tissue was immediately placed in phosphate-buffered saline (PBS, pH 7.4) and, within an hour, sectioned into strips, placed into cryovials, snap frozen, and stored in liquid nitrogen (−195 °C).

### 3.5. Ex Vivo Diffusion Studies

Pericardial tissue was thawed for 10 min in PBS, after which the tissue was sectioned into approximately 7 × 1 cm^2^ each per experiment. Tissue sections were mounted in PermeGear in-line flow-through diffusion cells (exposed area 0.039 cm^2^), part of a PermeGear 7-in-line flow-through diffusion system (PermeGear Inc., Hellertown, PA, USA). The tissue sections were equilibrated for 10 min in PBS (pH 7.4) containing 0.2% (*w*/*v*) SLS at 37 °C in both the donor and acceptor compartments of the diffusion cells before each permeability experiment started. PBS in the various donor compartments was replaced with 1 mg.mL^−1^ equivalent concentrations of BDQ-loaded nanoparticles. PBS containing 0.2% (*w*/*v*) SLS was pumped through the receptor chambers at a rate of 1.5 mL.h^−1^ at 37 °C, and samples were collected using a fraction collector at 2 h intervals for 24 h. The study was repeated three times. After collection, samples were diluted with methanol/acetonitrile (85:15) at a 50:50 ratio and analyzed by HPLC. Cumulative amount (µg.cm^−2^) versus time graphs were constructed, and the steady-state flux rates (µg.cm^−2^.min^−1^), as in Equation (4), and lag phases (min) were obtained from the linear portion of the slopes of the cumulative curves and the x-axis intercepts, respectively. The apparent permeability coefficient was calculated from the steady-state flux as in Equation (5).

(4) $$J=Q/\left(A\;\times \;t\right)\;$$

(5) $$P=\frac{J}{\left(\mathrm{Cdoner}-\;\mathrm{Creceptor}\right)\;}$$

where

J is the steady-state flux, Q is the quantity of substance crossing the membrane (in µg), A is the membrane area exposed (in cm^2^), and t is the time of exposure (in min).

P is the apparent permeability coefficient, Cdonor is the concentration in the donor compartment of the flow-through cell, and Creceptor is the concentration in the receptor compartment of the flow-through cell. The experiment was conducted under sink conditions and infinite dosing, and as such, Cdonor ≈ Creceptor.

### 3.6. Tissue Integrity Studies

#### 3.6.1. Transepithelial Electrical Resistance

Tissue sections were inserted onto Transwell membrane inserts (6.5 mm, 0.4 μm, 0.33 mm^2^) and 0.1 mL PBS was added to the apical side, while 0.6 mL PBS was added to the basolateral side into the culture wells. A Millicel^®^ ERS-2 hand-held resistivity meter (Billerica, MA, USA) was used to measure the tissue resistance of membranes before and after permeability experiments.

#### 3.6.2. Fourier Transform Infrared (FTIR) Spectroscopy: Tissue Integrity Studies

ATR-FTIR was also used in conjunction with resistance tests to check the tissue integrity of the pericardium before and after 24 h of the ex vivo permeation experiment. Dried samples of porcine and human pericardia (before and after testing) were analyzed at 110 psi pressure in a range of 4000–650 cm^−1^ over 20 scans to obtain relevant spectra.

### 3.7. Statistical Analysis

An unpaired Student *t*-test with Welch’s correction was utilized to compare possible differences between the steady-state flux values and apparent permeability constants of bedaquiline across the human and porcine pericardial tissues, using a 5% significance level.

## 4. Conclusions

The bedaquiline-loaded, mannan–chitosan bi-polymeric nanosystem was successfully synthesized. The size was verified to be mostly between 100 and 200 nm using both a zeta sizer and SEM. The drug showed complete encapsulation into the nanosystem, as per SEM and FTIR, and encapsulation efficiency. Mannan enhanced the gelling ability of the nanosystem, with further properties to ensure targeted delivery. The excellent permeation and release of the nanosystem ex vivo as compared to in vitro dissolution studies is possibly due to biofactors such as enzymatic degradation from the snap-frozen ex vivo tissue. Enzymatic hydrolysis of chitosan by lysosomes, an organelle containing proteolytic enzyme found in pericardial cells, may therefore improve the drug release across the pericardium, as chitosan is prone to enzymatic biodegradation in the body. The tissue integrity was maintained; however, more studies need to be carried out to confirm the effect of the nanosystem on the barrier. While mannan-mediated targeting of antigen-presenting cells presents a promising approach, further biodistribution studies are required to confirm selective accumulation in cardiac or pericardial tissues. Additionally, further studies are required to confirm biocompatibility, including in vitro cytotoxicity assays and in vivo safety profiling, before progressing to translational or clinical stages. Results from the study indicate prospects of bedaquiline’s ability to permeate across the pericardium as compared to the standard anti-tuberculosis treatment against tuberculous pericarditis. Unlike existing BDQ nanoformulations aimed at pulmonary delivery, this study presents a cardiac-specific delivery system utilizing mannan-functionalized COS nanoparticles to achieve enhanced permeation across pericardial tissue. This disease targeted and localized approach may offer a therapeutic advantage in treating drug-resistant TB pericarditis.

## Figures and Tables

**Table 1:** Transepithelial Electrical Resistance Measurements Across Porcine and Human Pericardial Membranes Before and After Diffusion Studies. Data represents the mean ± SD from three independent experiments, each with three technical replicates.

Mean Electrical Resistance Ohm (SD)	Human Pericardium (n = 3)			Porcine Pericardium (n = 3)		
	(i)	(ii)	(iii)	(i)	(ii)	(iii)
Before diffusion study	150.67 (±4.64)	166.33 (±5.43)	153.00 (±5.10)	147.33 (±4.99)	151.00 (±2.16)	138.33 (±5.25)
After diffusion study	147.67 (±6.24)	163.00 (±2.16)	146.33 (±4.50)	152.00 (±2.83)	159.00 (±2.16)	131.67 (±5.25)
Difference (%) Mean % difference	1.99 (±1.06)	2.00 (±1.97)	4.36 (±0.39)	3.17 (±1.47)	5.30 (±0.00)	4.82 (±0.00)
	2.78 (±1.14)			4.43 (±0.49)		

## Data Availability

The authors declare that the data supporting the findings of this study are available within the paper. Should any data files be needed in any other format, they are available from the corresponding author upon reasonable request.

## Abbreviations

The following abbreviations are used in this manuscript:

M.tb	*Mycobacterium tuberculosis*
EPTB	Extrapulmonary Tuberculosis
MDR-TB	Multidrug-resistant Tuberculosis
TBP	TB Pericarditis
BDQ	Bedaquiline
COS	Chitosan Oligosaccharide Lactate
NF-Κb	Nuclear Factor Kappa B
MAPK	Mitogen-Activated Protein Kinases
AMPK	Adenosine Monophosphate-Activated Protein Kinase
XDR-TB	Extensively Drug-Resistant Tuberculosis
MN	Mannan Oligosaccharide
IL	Interleukin
IFN	Interferon
TNF	Tumor Necrosis Factor
PBS	Phosphate-Buffered Saline
SLS	Sodium Lauryl Sulphate
HPLC	High-Performance Liquid Chromatography
DCM	Dichloromethane
sTPP	Sodium Tripolyphosphate
MWCO	Molecular Weight Cut-Off
PDI	Polydispersity Index
SEM	Scanning Electron Microscopy
FTIR	Fourier Transform Infrared
XRD	X-ray Diffraction
TGA	Thermogravimetric Analysis
MIC	Minimum Inhibitory Concentration
ATT	Anti-Tuberculosis Treatment
RP-HPLC	Reverse-Phase High-Performance Liquid Chromatography
SD	Standard Deviation
